# “It Happens, But I’m Not There”: On the Phenomenology of Childbirth

**DOI:** 10.1007/s10746-021-09585-4

**Published:** 2021-05-27

**Authors:** Dylan Trigg

**Affiliations:** Institut Für Philosophie (Room 1020), University of Vienna, Sensengasse 8, 1090 Wien, Austria

**Keywords:** Childbirth, Embodiment, Phenomenology, Anxiety, Pregancy, Feminism

## Abstract

Phenomenologically grounded research on pregnancy is a thriving area of activity in feminist studies and related disciplines. But what has been largely omitted in this area of research is the experience of childbirth itself. This paper proposes a phenomenological analysis of childbirth inspired by the work of Merleau-Ponty. The paper proceeds from the conviction that the concept of anonymity can play a critical role in explicating the affective structure of childbirth. This is evident in at least two respects. First, the concept of anonymity gives structural specificity to the different levels of bodily existence at work in childbirth. Second, the concept of anonymity can play a powerful explanatory role in accounting for the sense of strangeness accompanying childbirth. To flesh these ideas out, I focus on two attributes of birth, sourced from first-person narratives of childbirth. The first aspect concerns the sense of leaving one’s body behind during childbirth while the second aspect concerns the sense of strangeness accompanying the first encounter with the baby upon successful delivery. I take both of these aspects of childbirth seriously, treating them as being instructive not only of the uniqueness of childbirth but also revealing something important about bodily life more generally. Accordingly, the paper unfolds in three stages. First, I will critically explore the concept of anonymity in Merleau-Ponty; second, I will apply this concept to childbirth; finally, I will provide an outline of how childbirth sheds light on the broader nature of bodily life.

“It happens, but I’m not there”: On the Phenomenology of Childbirth^1^

I have no more awareness of being the true subject of my sensation than I do of my birth or my death.

Merleau-Ponty, *Phenomenology of Perception*

## Introduction

Phenomenological research on the topic of childbirth and pregnancy is currently thriving in a number of ways. Whether it be the anxiety surrounding the birth process (see Staehler 2016), the sense of the pregnant body as alienated and doubled (see Young 2005), or the notion of birth and pregnancy as an opportunity to re-establish divisions between self and other (see Heinämaa 2014), phenomenology has evinced an enduring interest in childbirth and pregnancy not only as a key event in human experience, but also as a source of insight for the structure of bodily life more generally (see Bornemark and Smith 2012; Stone 2019).

One of the key strengths of a phenomenological approach to childbirth and pregnancy is that the method avoids the pitfalls of delineating the salient features of childbirth in a predetermined fashion. Problematizing the notion of childbirth as a passive experience, a site of euphoric bliss, or a process of animalistic regression, phenomenology accents the ambiguity and multiple levels of meaning structuring pregnant embodiment, in the process registering how birth can both integrate and disintegrate maternal subjectivity equally (see Söderbäck (2018, 2019; Welsh (2013).

Nevertheless, while there has been a surge of interest in maternal embodiment from a phenomenological perspective, what is missing from the current research on birth is a sustained focus on what author Maggie Nelson called the “profoundly strange” aspect of childbirth. While there are exceptions to this omission (see Staehler 2016), the theme of strangeness is nevertheless underresearched within the literature. The aim of the current paper is to contribute to this research lacuna by proceeding from the conviction that it is beneficial to centralise strangeness in at least two respects. First, the mood of strangeness issues a challenge to the notion of birth as irreducibly horrific or otherwise ineffably miraculous. Both of these trajectories risk eroding the specificity of childbirth and potentially stigmatizing painful childbirths. Moreover, on a conceptual level, the idea of strangeness registers the ambiguous structure of childbirth as involving disparate and often conflicting dimensions. This is manifest not only at a conceptual level, but also on an affective level insofar as birth often involves a sense of the body as one’s own and alien concurrently.

The hypothesis for this paper is that the strangeness of birth is best captured through the concept of *anonymity*. There are a number of ways to think about the role anonymity plays in childbirth and pregnancy (see Bornemark 2016). In the present paper, I propose to think of the relation between anonymity and birth in terms of the uncanny terrain between the body as one’s own and the body as other. To speak of the body in its otherness invokes the idea of the pregnant body as being the host of another body. In this light, we might be tempted to think of the pregnant body as the host of another life, or, the pregnant body as a perceptually foregrounded object of pain. More enigmatic than this, however, my focus in this paper is the body-in-labour as a body that brings to light the inheritance of an anonymous and immemorial temporality outside of lived experience. In framing the anonymity of the body in this respect, I proceed from the point of view that the anonymous and immoral aspect of subjective life presents a challenge to the idea of embodied subjectivity as being univocal and integrated.

To unpack this claim, I will examine two salient aspects of childbirth, which bring the tension between anonymity and birth into vivid detail. The first aspect concerns the sense of leaving one’s body behind during childbirth, as reported in women’s narratives of labour (Akrich and Pasveer 2004). Typically, these reports are marked by a sense of alienation from the body as one’s own. The body is experienced in its thinglike status as undergoing a process, which the labouring woman is witnessing in partial separation. The second aspect of childbirth that I will concentrate on concerns the sense of strangeness accompanying the first encounter with the baby upon successful delivery. What characterises these experiences is a profound sense of disbelief that the baby is actually here, despite the baby being empirically perceivable. My source material for both of these aspects is a series of first-person narratives documented in existing research (Akrich and Paveer 2004; Lupton and Schmied 2013). I take both of these aspects of childbirth seriously, treating them as being instructive not only of the uniqueness of childbirth but also revealing something important about bodily life more generally, especially in terms of where the body as my own begins and ends. Such dimensions are usually taken-for-granted in everyday experience, and thus they raise questions concerning the integrity of selfhood and the role multiple levels of bodily life play in generating different affective responses.

Methodologically, my aim in this paper is to develop the concept of anonymity as it has been conceived in the work of Merleau-Ponty. The concept of anonymity in his thought involves several strands; and like his thought as a whole, the concept is worked and reworked on, refined and radicalized as his thought progresses toward its endpoint. More than this, the theme of anonymity comes under different terms: as prepersonal or impersonal, “an original past,” natural time, a time without a subject, an “absolute past of nature,” “primordial silence,” “prehistory,” “a past which has never been present,” and, in his later work, “the Memory of the world” (Merleau-Ponty 2012: 137/160, 184/214, 240/277, 252; 1969: 194). In each of these variants, the concept of anonymity, as I will explicate shortly, denotes an impersonal structure of perception that renders perceptual life possible in the first place.

My point of departure for applying this concept to birth begins from the conviction that Merleau-Ponty’s account of anonymity tends to privilege themes of integrity and synthesis while neglecting how anonymity can serve as a threat or rupture to the unity of selfhood, especially in the context of limit-experiences. One might say, in this sense, that the subject of Merleau-Ponty is a particular kind of subject, at least in how it has been interpreted in Anglo-American scholarship. It is a subjectivity marked by a felicitous orientation toward others, hinging at all times on a bodily sense of an I-can rather than an I cannot, and framed by an affirmative relationship to ambiguity. With the concept of anonymity as my foundation, the paper proceeds in three stages. First, I will critically explore the concept of anonymity in Merleau-Ponty; second, I will apply this concept to childbirth; finally, I will provide an outline of how childbirth sheds light on the broader nature of bodily life.

## The Concept of Anonymity in Merleau-Ponty

The genesis of Merleau-Ponty’s concept of anonymity is motivated by a desire to account for the structure of perception, where perception is governed by a field of meaning and sense, unifying temporality into an overarching arc of significance. The point of departure is a question that runs throughout his work; namely, *who is it that perceives*? We generally respond to this question in a self-affirmative way. It is *I* who perceives the lighthouse appearing from the fog; it is *I* who type on the keyboard, and it is *I* who walk to the door a few steps away. I grasp things, I solicit memories from experience, and anticipate events that may or may not come to fruition. I look at my own body; it is a mass of space organised into parts, and these parts carry with them an affective energy that is seized with an atmosphere of ownership. Suffering from sickness, I feel my body dissent from me. During moments of fatigue, my body has gone astray. When it returns to me, then I simultaneously return to myself, to that place where I feel at home in an otherwise uncertain world.

But who—or what—is this “I” that provides a restorative function in the world? Who is it that enables me to function in the world, to institute meaning, and integrate a bodily mass of organs into a synthesis capable of walking from one corner of the room to another, or, in the case of the female body, of giving birth to another life? And who, above all, is the subject of perception? It is a question that compels Merleau-Ponty’s thought forward.

In *Phenomenology of Perception*, this question will be met with several responses. Central to each of them is the notion of an “organic thought” that mediates between the personal and the impersonal, the physical and the psychical. The notion of an organic thought captures Merleau-Ponty’s insistence on how human existence is characterised by an overarching sense of unity, subtended to at all times by a prepersonal level of corporeal perception, which serves to both cohere the past into the present while at the same time developing an *intentional arc* that establishes the “unity of the senses” (Merleau-Ponty (2012: 137). Merleau-Ponty puts the issue as follows: “Prior to stimuli and sensible contents, a sort of inner diaphragm must be recognised that…determines what our reflexes and our perceptions will be able to aim at in the world, the zone of our possible operations, and the scope of our life” (2012: 81). This prepersonal—or, *impersonal*—inner diaphragm determines not only a highly complex relation to the world, but also the levels and sub-levels of human subjectivity. Indeed, it is thanks to the body in its anonymity and generality that the personalised “I” is able to be situated in the world in the first instance.

For Merleau-Ponty, the organisation of the body is not reducible to thematic experience but instead hinges at all times on another layer of intentionality that renders thematic experience possible in the first place. These “regions of silence [which] are marked out in the totality of my body” generate an ambiguous depth in his phenomenology of the body, ambiguous not only in the sense of being a particular kind of object, but also in the sense of never being entirely possessed by the subject, both temporally and spatially (Merleau-Ponty 2012: 84). In an especially critical passage, he writes as follows:

A margin of *almost* impersonal existence thus appears around our personal existence, which, so to speak, is taken for granted, and to which I entrust the care of keeping me alive. Around the human world that each of us has fashioned, there appears a general world to which we must first belong in order to enclose ourselves within a particular world ... my organism—as a pre-personal adhesion to the form of the world, as an anonymous and general existence—plays the role of an *innate complex* beneath the level of my personal life. (Merleau-Ponty 2012: 86)

This peculiar structure of an “*almost* impersonal existence” that keeps me alive renders the body a double-sided entity. Just as it reveals itself to me as an expressive and irreducibly personalised body, so it simultaneously evades me. We are subjected to our bodies in a literal way, as Merleau-Ponty has it, “my life is made up of rhythms that do not have their *reason* in what I have chosen to be” (Merleau-Ponty 2012: 86). Critically, the impersonal existence that forms an arc in and around personal existence only does so *partially*. Thus, that Merleau-Ponty speaks of an “*almost* impersonal existence” is worth noting (2012: 86). As an “*almost* impersonal existence,” my body is never entirely anonymous but nor is it unquestionably singular; rather, it is a strange hybrid of rhythms, habits, and processes that are conceived in the midst of finite life and which simultaneously belong to an older order of Nature. “Personal existence,” Merleau-Ponty writes, “is intermittent,” and behind the surface of being a discrete self who is identifiable with “one’s own” body, there exists another kind of existence, elemental and indifferent to the self that assumes a relationship to it (2012: 86).

Merleau-Ponty’s concept of the anonymous body has been met with a divergent set of responses. For some scholars, like Shannon Sullivan and Elizabeth Grosz, the concept of the anonymous body leads to an effacement of difference, and even an ethics of domination (see Grosz 1994; Sullivan 2008). For other scholars, the idea that Merleau-Ponty’s concept of anonymity negates difference reflects a basic misunderstanding of his position; namely, that difference is itself underpinned by and predicated on a prior notion of anonymity (Burke 2013).

Alongside these reflections, there is another potential problem in Merleau-Ponty’s thought; namely, that he tends to overlook how impersonal dimensions of existence are given to experience, either directly or indirectly, in an affective *sense*, especially when the affective tonality is disruptive in nature. For the most part, his thought is grounded in a principle of integration, such that the different levels of perceptual life fold into one another without any fault lines developing in the process. In Merleau-Ponty’s defence, it might be asked why impersonal and anonymous aspects of human existence might be conceived as a figure of threat in the first instance? After all, much of our waking life involves an implicit trust in the body without the need to interrogate what relationship the body in its generality and anonymity. The body is not, however, a homogenous unit of experience and the relationship we have to different aspects of its existence is contingent on a multiplicity of factors not least the affective situation we find ourselves in. In moods such as anxiety and in situations such as heart attacks, anonymity presents itself not as an innocuous structure of lived experience but an aspect of existence that serves to destabilize, threaten, and estrange the image we have of who we are. Nowhere is this tension clearer than in childbirth.

## The Body Takes Over

What we have seen in Merleau-Ponty is an account of the body that involves several levels of intentional, temporal, and perceptual existence functioning in tandem. What I would like to do now is begin the process of applying this conceptual landscape to the specific instance of childbirth. Indeed, as we will see, childbirth presents an especially vivid articulation of the multiple levels of ambiguity at work in Merleau-Ponty. As intimated above, I am concerned here with how Merleau-Ponty can help us understand two aspects of birth: (1) The sense of the body as taking over when giving birth; (2) the sense of strange wonder upon the first encounter with the baby at the moment of birth.

In each of these moments, labour is not inaugurated through personal intervention, but rather through waiting for the body to begin of its own accord. Levesque-Lopman, one of the first feminist philosophers to offer a first-person account of childbirth, writes: “Once labor has begun it continues independent of the will. It is as if a woman’s body is being taken over by a force. She cannot will the onset of labor, nor can she consciously or intentionally alter its pattern once it has begun. It proceeds inexorably to its end regardless of her desires” (Levesque-Lopman 1983: 267). Once underway, the woman in labour becomes implicated by the rhythm of her body, caught up in a process in which she has little choice but to submit to. As such, the start of labour from a bodily level does not necessarily coincide with the start of labour from an experiential perspective.

This is not only evident in the temporality of labour, but also in the sense that the body-in-labour is to some extent indecipherable. From a first-person perspective, establishing what is going on in and with the body is not always obvious. Contractions can be misread as cramps and cramps can be misread as contractions. Far from a transparent surface, then, the body presents itself as a depth to be interpreted and deciphered. When these processes are then “explained” through the intervention of medical practitioners, the result is not necessarily clarification but instead alienation grounded in the objectification of the body (See Young 2005). Of course, the extent to which this sense of alienation provokes discomfort varies widely, as Welsh remarks, “[f]or each woman, this sense of alienation is naturally different given the context of her pregnancy, her physiology, and her psychical state; however, it is similar in all women given the presence of this alien being” (Welsh 2008: 53). As the course of labour develops, the sense of the body as alien escalates and one way this is evident is in terms of being swept away by the sheer force of the body-in-labour. Several quotes here give us a sense of this.

The sensations are strong, overpowering, and can be at the same time delightful. One is swept away like a little boat at sea in a great storm of exultant emotions and a tremendous sweep of physical energy. The body takes over in what seems a wholly marvellous way … We can only be in awe and deliver ourselves over, in faith, to this wonderful thing—the female body at the work of creation. (Kitzinger 1987: 201)

At about 8.30 I had a contraction I couldn’t really manage. My body wanted nothing but to push. I couldn’t help but push three or four times … For a moment I was completely freaked out by this violence in me. I didn’t realize this was a pushing-contraction and that this was the last phase of the birth. (Carole cited in Akrich and Pasveer 2004: 72)

Before [the epidural], between two contractions I couldn’t rest, in fact I panicked, when I felt a contraction coming on, I panicked. After the epidural I could enjoy the moment more … Because it’s true that every contraction, I was scared every time, I was really stressed, and with the breathing, I wasn’t managing too well, I really had to force myself to concentrate on my breathing…I wasn’t comfortable. (Annie cited in Akrich and Pasveer 2004: 73)

We have varying interpretations of the same process. In each case, there is a characterisation of the body as having an agency and teleology of its own, at least partially, and which is to some extent independent of the individual. In the first report, by Sheila Kitzinger, the climax of labour is presented in near symphonic terms, as a sublime crescendo, in which all the parts converge in a seamless whole. The aesthetic dimension of this account is framed not only by the language of awe, with its connotations of sublimity, but also by the distinction between we who are bystanders to this event and the female body itself, which takes over in a seamless way. Throughout, there is a trust in the body as benevolent and being on the side of the subject. As Kitzinger has it, submitting to the body as a “thing” does not result in alienation or anxiety, but instead an enhanced sense of integrity.

In the remaining accounts, each sourced from research by sociologists Akrich and Pasveer concerning the experience of embodiment and disembodiment in childbirth, the seamless integration between one’s own body the body as a site of independence is augmented with a divisive and fragmented account of the labouring body as disintegrating the living subject. In each account, the language of awe and wonder is replaced with a vocabulary of trepidation and fear. Throughout this process, the sense of agency is not eliminated but instead amplified, as Akrich and Pasveer observe, “[p]aradoxically, it is when the ‘body-in-labour’ imposes on her body a demand that she cannot ignore that she recovers a maximal ‘agency’” (2004: 73). Yet what Akrich and Pasveer overlook is that the recovered agency is not strictly that of the familiar “I” but instead an augmented fusion of familiar and unfamiliar aspects of identity inhabiting the same space. In the final narrative from Annie, this confrontation with a strange variant of the embodied-self results in a sense of acute panic. Between contractions, Annie reflects, “I couldn’t rest, in fact I panicked, when I felt a contraction coming on, I panicked” (Annie cited in Akrich and Pasveer 2004: 73). Akrich and Pasveer correctly note that there are two narratives at work here, “the contractions (of the uterus) and the ‘I’ of the narration” (2004: 73). Unlike the previous cases, for Annie, the multiple narratives are not consolidated into a unified whole but instead present themselves as mutually opposing forces. The result of this inability to preserve integrity is *panic*.

At stake in this panic is not simply an intolerance of pain. To reduce the sense of fear and anxiety in these accounts to an intolerance of pain would be to overlook the broader significance. Beyond the issue of visceral pain, what is also at stake here is a panic concerning the advent of a body experienced as partially other. Indeed, the “pain” involved is not only a pain grounded in physical discomfort, it is also an anguish rooted in the anxiety of a body that is no longer one’s own. The dualism here between multiple narratives is not a form of Cartesianism, but instead an affirmation of the self as fundamentally and irreducibly *embodied*. Indeed, it is precisely because the labouring woman is an embodied woman that the disruption of this structure causes anxiety rather than abstract curiosity. Annie’s “discomfort” is grounded in what Akrich and Pasveer term a “protective dissociation” (2004: 73). In effect, the division between the body as taking over and the “I” that remains in place is too discordant to be reconciled. In this respect, the introduction of the epidural is notable because it effectively “blurs” the boundary line between the body-in-labour and the embodied self, such that there is a partial reconstitution of these joint structures. “After the epidural,” Annie remarks, “I could enjoy the moment more,” and this level of enjoyment is predicated on a benevolent dissociation whereby the autonomy of the body is accepted rather than resisted. In a passage from another childbirth narrative, this sense of the body as *other* is rendered in vivid detail. In a narrative from a woman called Myriam, also sourced from the critical research of Akrich and Pasveer, we are told the following:

At one point Ria [the midwife] told me to feel the head but I didn’t want to do that. She thought that it might make me push more. She took my hand and wanted to let me feel it. I shouted I did not want it. Hans [the husband] told Ria not to do it if I didn’t want to. I found that a bit creepy. I didn’t want to see a mirror or anything. But she insisted even when I told her I did not want to feel the head. Then she took my hand and I found that creepy. (Myriam cited in Akrich and Pasveer 2004: 76)

Central to this narrative is the role the intermediary figure of the midwife plays in contributing to the objectification of the body as a thing. As Akrich and Pasveer note, the intervention of medical practitioners generates a “tension between the body-in-labour—the object of the different participants’ attention—and the woman’s ‘own’ body” (Akrich and Pasveer 2004: 76). The result is the materialization of the body in its “*creepiness*”. There are several reflections to be made on the inclusion of the term “creepy” here. The first point to note concerns the objectification of the labouring body. The hands and head that appear in this vivid description do so not as integrated sectors of a living being but as discernible objects that are foregrounded in their strangeness. Despite resistance, the hand of Myriam is taken by the midwife and directed toward the head of the emerging infant. That this process unfolds against her wishes only reinforces the sense of the labouring body as a thing to be observed by the labouring woman, as Akrich and Pasveer remark, “the woman is in and out of her body, both actor and spectator” (Akrich and Pasveer 2004: 76).

The consequence of this felt dualism is a sense of uncanniness directed toward both the mother’s hand and the baby’s head. In this respect, it is striking that the term “creepy” appears twice in this narrative. With its connotations of dread and uncanniness, the term is apposite. What is creepy is the sense of an irreducibly familiar aspect of one’s body rendered partially foreign and thinglike. In the hands of the midwife, the hand that is ordinarily a tacit dimension of lived experience has now become an instrument employed to measure the distance of an emerging head. In effect, the hand and the head have both become animated by a force anterior to the labouring woman.

Here, one cannot help but think of Freud’s account of the uncanny as focusing on phenomena such as “a hand cut off at the wrist, feet which dance by themselves” (Freud 2003: 150). Without subscribing to the psychosexual infrastructure guiding Freud’s analysis, the observation that the body gains an agency of its own is central to the creepy affectivity described in the narrative above. As Tanja Staehler has noted, birth’s uncanniness is manifold, but its central manifestation is grounded in the sense of the body as undergoing a process that is in some sense autonomous and unimaginable (2016).

Throughout this, Myriam has not disappeared, nor has she left the scene. At no point in this account is there an erasure of “mineness” or “ownness” (Husserl 1960). Far from this, she is in fact vividly present within the scene, evident in the usage of phrases such as “I shouted,” “I didn’t want to,” and “I found that creepy”. Thus, if the personalised “I” retains its centrality within the event of labour, then there is nevertheless an experience of anonymity offsetting the personal subject. Indeed, it is precisely because ownership and mineness remain intact that the experience of the lived body as anonymous causes anxiety. It is, in the end, *my* body that is experienced as strange rather than *a* body or *the* body.

To speak in this sense of the subjective experience of anonymity might sound odd, given that an experience of an impersonal anonymity is already to personalise it. However, this tension is resolved so long as we remain heedful of Merleau-Ponty’s observation that an “*almost* impersonal existence” surrounds our personal existence. As an *“almost* impersonal existence,” we are never truly identifiable with our bodies if we take our body as a composite of singular and anonymous, contemporary and immemorial aspects working in tandem. Rather, “my life is made up of rhythms that do not have their *reason* in what I have chosen to be, but rather have their *condition* in the banal milieu that surrounds me” (Merleau-Ponty 2012: 86). For the most part, these rhythms are tacit and such reflections on anonymous life are absent in lived experience; carnal life is given to experience in and through the lens of a personal sphere, which provides an atmosphere of familiarity and constancy not only to the body but to the surrounding world more generally.

Yet atypical affective and clinical situations present a challenge to this familiar atmosphere. If labour reveals the limits of the body as one’s own, then it does so only because it retains a constellation of contradictory aspects inhabiting the same space: the body is both foreign and intimate, the world is as familiar as it is alien, and the sense of self is both integrated and fragmented in the same measure. In this respect, the experience of anonymity is thus an experience of being on the verge of a depersonalized and raw materiality without ever dissolving completely into it. This structure is given sharp thematic expression in the language of creepiness employed in the narrative above. The creepy body is a body that defies the story told of who one is, and, as such, has disclosed itself as an object resistant to integration.

## One Perceives

We have seen that labour can establish an opaque relationship to the body, such that the woman in labour can experience her own body as an organism undergoing a process that she herself is partially distanced from. We have also seen that this sense of the body as doing its own thing can lead to either a sense of exultation or panic. In order to grasp the affective tonality at stake in this movement of the one’s body becoming anonymous, it is necessary here to return to a question central to Merleau-Ponty’s thought; namely, *who is it that perceives*? As we saw above, Merleau-Ponty’s response to this question is complex and the account of embodied subjectivity that he posits is comprised from a multiplicity of different levels of bodily existence. Central to this is an anonymous modality of “*almost* impersonal existence” that subtends to personal life without ever being identifiable with it (Merleau-Ponty 2012: 86). In Merleau-Ponty’s terms, we entrust our personal existence to an “innate complex,” which effectively keeps us alive through operating on a latent level. Given this framework, Merleau-Ponty issues a challenge to the idea that perception is irreducibly one’s own, stating that “[e]very perception takes place within an atmosphere of generality and is presented to us as anonymous [and] if I wanted to express perceptual experience with precision, I would have to say that *one* perceives in me, and not that I perceive” (2012: 223). These reflections on the nature of perception do not concern simply the structure of sensation, but instead hinge on the experience of oneself as being involved with “another self that has already sided with the world” long before I myself as an ego have conceptualised that existence or even experienced it (2012: 224).

For the most part, these zones of bodily existence remain integrated into a unified whole, such that the anonymous body does less to impinge upon personal life and more to ground and support it. The beating of the heart, to take an example, is not something that I volitionally will of my own accord; but is instead a process that I resume and animate in and through my singular existence. As such, to say that I am the “author” of the beating of my heart is inaccurate. My heart beats in and through me as a “general existence [which is] destined to a physical world” (Merleau-Ponty 2012: 224). Registering that entire zones of bodily life are anonymous, impersonal, and immemorial means in the same measure recognising that “I never have an absolute possession of myself by myself” and, as a result, that “the anonymity of our body is inseparably both freedom and servitude” (Merleau-Ponty 2012: 87). Processes such as the beating of the heart, being able to perceive different shades of colour, and walking from one room to another are all situations in which the synthesis of bodily life reinforces the unity of subjectivity.

This integrative structure is evident already in several of the narratives describing birth, as Levesque-Lopman writes: “My body seemed to take over in a tremendous sweep of physical energy … As I tuned into the rhythm of my body, I had no doubt and my husband could only be in awe as I surrendered to the power of my body” (Levesque-Lopman 1983: 267). In descriptions such as this, there is an attunement between the singular and the general with each level of bodily existence working in tandem to engineer a specific end. In this respect, the metaphors of oceans and waves that emerge time and again in literary and clinical accounts of childbirth tend to be predicated on the image of “riding the wave,” such that there is an alignment of one’s own bodily rhythms to those rhythms which both precede and outlive us.

Here, Sara Heinämaa writes aptly: “The functions of pregnant and nursing bodies resemble the anonymous operations of our perceptual organs in providing a foundation for certain kinds of experiences” (2014: 40) However, Heinämaa proceeds to make a distinction between the anonymous body, as it operates in everyday experience, and how it then unfolds during pregnancy and labour. In the case of the former, the materialization of the anonymous body only comes to the foreground in cases of abnormal experience, such as pain and discomfort, and thus presents them as threats that potentially destabilize normative structures (Heinämaa 2014: 40f.). For Heinämaa, pregnancy—and by implication, labour—unfold differently insofar as they “contribute to the establishment of another norm” through being guided by a specific teleology (2014: 41).

However, not all accounts of childbirth conform to the idea of birth and pregnancy as generating a new sort of normative bodily practice. In other cases, the sense of the body as a form of anonymous resistance establishes a radical discord that contributes to a partial erosion of selfhood. In her analysis of childbirth, Jonna Bornemark puts the point as follows:

The movement of life is violent and doesn’t really care about me, i.e. about that already constituted subjectivity. It breaks up and redraws the limits, and in this way creates new subjectivities. New forms are shaped, new distinctions are made, and new borders are drawn. In the midst of this violence there is still a small room left for a subjectivity that can think: ‘This will end. There will be time again.’ My subjectivity is not fully erased, but it is drawn toward its limits. (2012: 268)

Bornemark captures the sense of the personal subject as neither wholly absent nor effaced by this advent of the body in labour, and it is precisely because there is a preservation of these two distinct levels of existence coming into sharp contact with one another that the event of birth entails a strange tonality that can easily border on anxiety if not panic. Contra Heinämaa, this recognition that our origin lies outside of ourselves does not necessarily “contribute to the establishment of another norm” but displaces the centrality of the personal self. Benevolent or not, this sense of another agency speaking through the human body has implications for how we understand ourselves.

The critical point to note here is that the relation between different levels of bodily existence is not simply a structural relation nor is it devoid of affect, but instead one that is laden with affective significance, and which can teeter between anxiety and awe. Such a point is already anticipated in Merleau-Ponty when he speaks of the pre-human and inhuman world as “hostile and foreign…no longer our interlocutor but rather a resolutely silent Other” (2012: 336). As a dimension of bodily existence, anonymity is without a face, silent, and yet it speaks through us; indeed, it employs the human body as its vehicle of expression. Concerning this corporeal manifestation of anonymity, Kristeva writes as follows in relation to pregnancy, “[w]ithin the body, growing as a graft, indomitable, there is an other. And no one is present, within that simultaneously dual and alien space, to signify what is going on. ‘It happens, but I’m not there.’ ‘I cannot realize it, but it goes on.’ Motherhood’s impossible syllogism” (1980: 237). Here, Kristeva locates the tension central to labour and pregnancy. *It happens, but I’m not there*: this formulation brings to light the strange sense of the I as being both present and absent in a process the labouring woman herself is only partly in control over. *It* goes on through her, without *it* ever being reducible to the *I* which gives expressive form to the process.

## Where Did This Baby Come From?

These accounts of birth as involving a sense of the anonymous body as taking over, leaving the personalised subject merely one aspect in a larger scenario, establishes a strange tonality to childbirth. Upon birth, this atmosphere of strangeness is not expired but instead retained in the immediate aftermath of the baby’s delivery. Merleau-Ponty himself notes this in his lectures on child psychology, writing that “[w]e often note that right after the birth, a sentiment of strangeness, of unreality arises” (2010: 80). One way this “sentiment of strangeness” manifests itself is in a question one finds in the literature; namely, *where did the baby come from*? Of course, the question is not a factual or empirical one concerning the different phases of birth, much less an appeal to Immaculate Conception; rather, the question is rooted in a strange disbelief that the baby is finally here. Several case studies, sourced from sociological research by Lupton and Schmied, attest to this peculiar moment.

The midwife handed her straight to me and I held her, but I had held her for a while, I just was – it was like looking at her and wondering ‘*Where did this baby come from*?’ You know, despite what I’d gone through, it was hard to associate that she was actually mine and she was out of my stomach ... Even holding her for the first few minutes are just, it wasn’t like she was mine, my kid, which is weird ... when you think of what you went through, it was really quite strange. (Tess cited in Lupton and Schmied 2013: 834)

Oh, I was just overcome, like, ‘*Where did it [the baby] come from?*’ My support people both laughed at me later one because they said ‘You just, like it was as if like, wow, I didn’t know that was going to come out!’ I didn’t know a baby was going to come out. It was just really spacey, a weird thing. (Kerry cited in Lupton and Schmied 2013: 834)

Where did the baby come from? There are several reasons for asking this strange question. In the first case, it is not obvious that the first encounter with the baby should entail unequivocal and straightforward affirmation. Given that there has been a lengthy gestation period, which has been framed by an inseparable alliance between the mother and her foetus, the eventual separation of these bodies carries with it a wide range of affective resonances. Beauvoir speaks in this respect of “an astonished melancholy in seeing [the baby] outside, cut off from her,” writing that “it is no longer an indistinct part of themselves but a piece of the world; it no longer secretly haunts the body but can be seen, touched” (Beauvoir 1989: 486, 490). Beauvoir highlights the strange metamorphosis undertaken between an intimate knowledge of the baby as an inner being whose flesh is figuratively and literally intertwined with the mother to a stranger who now faces the mother in a wholly unfamiliar way. As if from nowhere, the baby has a voice and a form, which is being seen for the first time. The encounter is uncanny insofar as it is framed by the disjunction between an inner world of familiarity offset with an exterior form that is entirely novel and thus unrecognisable.

But there is another context for the question of where did this baby derive from, and it is rooted in the specific narrative of labour more specifically. As we have seen, childbirth involves the interplay of personal and impersonal levels of bodily existence inhabiting the same space and time. This rapport between different levels of bodily and perceptual life unfolds in a dialectical manner. At times, there is a synchronicity of the body-in-labour with the woman-in-labour. At other times, these rhythms and temporalities disembark on a conflicting path, resulting in an alienation from the body in its anonymity and elemental force. As Lupton and Schmied write, “it was…very difficult for these women to conceptualise the notion that their own bodies had produced another body” (2013: 834). At the heart of this difficulty is not an intellectual deficit, but instead an affective resistance to the notion of one’s own body as undergoing a process that seemingly comes from beyond but is given thematic expression in a singular and intimate way.

In the immediate aftermath of birth, this dialectical interplay of differing and divergent levels of existence tends to re-integrate, a process framed by the radical contrast between the duration of labour and its eventual closure, and grasped affectively in terms of a profound sense of *relief*. Lupton and Schmied note that the expulsion of the body from the vagina is a relief both in the sense of a cessation of the physical sensations accompanying the birth process but also—and perhaps more critically—in terms of the recovery of the labouring body (Lupton and Schmied 2013: 833). Considering this dynamic more carefully, what is significant is that it is only upon the resumption of the I that there can be a retroactive grasp of what the labouring woman has just undergone. Up until this point, when the body is said to have taken over, there is an adjoining diminishment of subjectivity and thus a partial surrender to what Merleau-Ponty described as the other “subject beneath me, for whom a world exists before I am here, and who marks out my place in it”(2012: 296). We also recall here both Kristeva’s remark that “‘It happens, but I’m not there.’ ‘I cannot realize it, but it goes on’” (1980: 237), as well as Bornemark’s observation that “[m]y subjectivity is not fully erased, but it is drawn toward its limits” (2012: 268). Each of these claims points to the idea that in the midst of labour, the I has become depersonalised insofar as it has been stripped of its personal attributes and swept up in the primacy of the anonymous body. At all times, it is implicated by a course of action over which the labouring woman has little control.

Seen in this way, the immediate moment after birth is thus privileged, not only for its human value, but also because it involves a constellation of divergent modalities of life briefly occupying the same terrain. For a transitory moment, anonymity and singularity converge; the body-in-labour is experienced with radiant insight in all its strangeness before it is reconsolidated back into the living subject. “From the head-nodding coma of an endorphin-soaked dream,” Cressida Heyes writes, “I woke up, into the fullest and most alive state of alert presence … I was aware of every detail of the drama as my body split in two” (2012: 140). It is against this fleeting moment that the baby is handed to the mother, and she must now make sense of how it arrived while being caught up in the drama of a bodily existence that pushes subjectivity to a limit.

“It is strangely miraculous,” so Beauvoir writes, “to see and to hold a living being formed within oneself and issued forth from oneself. But just what part has the mother had in the extraordinary event that brings into the world a new existence? She does not know” (1989: 486). Beauvoir’s reflections are confirmed in a remark cited above, namely: “It was hard to associate that she was actually mine and she was out of my stomach”. There is little reconciliation here between inner and outer, between the irreducibly anonymous and impersonal and the overwhelmingly singular and personal. Holding one’s own baby is not enough to integrate it *as* one’s own, and, as a result, a sense of strange disbelief intervenes. Despite seeing with her own eyes that the baby has arrived, it is difficult to process this data except as an abstraction.

No doubt for this reason that the sentiment of strangeness accompanying childbirth is often twinned with a parallel sense of disgust, as a woman called Amanda notes: “When the baby had come out and they had put it on me all bloody, I said ‘Get him off me!’ ‘Sorry’, I said, ‘Can you clean him up?’ I just couldn’t—with all that blood it was just so disgusting. And I thought maybe when it came out and it was all bloody I’d really want to hold it, but I didn’t” (Amanda cited in Lupton and Schmied 2013: 834). This emotionally powerful response attests as much to the visceral materiality of birth and afterbirth as it does the symbolic significance blood and gore play in alienating the mother from the newborn. As a marker of an anonymous biological order, the presentation of the infant as raw matter underscores and amplifies the association of anonymity with a threatening presence. It is against this context that the impulse to “clean him up” gains a significant meaning, as Lupton and Schmied write, “[s]he only feels the desire to hold her newborn infant when his body has been cleaned and her disgust is thus abated” (2013: 834). To clean him up means to divest the newborn of is strangeness, effectively washing away the traces of the liminal place from where he came; to clean him up, in short, is to begin the long process of personalising and thus integrating the newborn as one’s child.

## Conclusion

The aim of this paper has been to develop a phenomenological account of childbirth that centralises the role anonymity plays in accounting for both the multiple levels of bodily ambiguity involved in the birth process as well as the tonality of strangeness accompanying this process. As a conclusion, it is worth explicitly pointing out (i) how this account advances current research on childbirth; (ii) how childbirth differs from related phenomena; and (iii) what insights this research can shed on a broader understanding of bodily life.

Let me take the first point. Until now, phenomenological research on childbirth has tended to focus on pregnancy, and especially on the ambiguous structure of pregnancy. What has been largely neglected in this pool of research is the conceptual and affective nature of childbirth as a specific event. In parallel, while there has been a steady stream of research on childbirth from a sociological perspective, what this disciplinary perspective has tended to overlook is the broader conceptual implications of this process. In response to this context, the present paper has sought to advance the understanding of childbirth in at least two critical ways.

First, I have attempted to develop the idea of childbirth as inherently ambiguous by employing the concept of anonymity as an explanatory device. The advantage of applying this concept is to generate both a fine-grained analysis of the multiple levels of bodily existence operational during childbirth but also to provide a more nuanced account of the affective structure of childbirth. On the first point, my focus on anonymity contributes to the idea of pregnant embodiment as ambiguous by attending to how the labouring body can be understood as both one’s own and alien in the same measure. In correspondence, this paper has also advanced the thesis that the affective tonality of childbirth is best captured as a form of strangeness. While other researchers have hinted at this, especially in the work of Kristeva and Stähler (see Kristeva 1980; Staehler 2016), the current paper has nevertheless advanced this field of research by developing the idea of “pain” not only as a physical attribute but also as a mode of anguish concerning the experience of one’s own body as anonymous.

These points are important not only because they offer a new perspective on childbirth, but also because they problematize the idea of childbirth as being unequivocally blissful or horrifying. In turn, one potential benefit of centralising the concept of anonymity and the adjoining theme of strangeness is to avoid stigmatizing narratives of birth that resist conforming to normative expectations. Indeed, as central to the birth process, strangeness and anonymity recognise the irreducible alterity of childbirth and its fundamental resistance to being domesticated to nothing more and nothing less than an “everyday event”.

Concerning what distinguishes childbirth from related phenomena, there are a number of ways to account for the specificity of childbirth as a unique event. To generate some clarity on this, let us take the example of a heart attack as a contrasting case study. On this point, I am indebted to the research of Kevin Aho and specifically to his recent phenomenological study of his own heart attack (2019). As with childbirth, a heart attack entails the modification of one’s lived experience of their body from a tacit centre of experience to an object scrutinised by both oneself and others (Aho 2019: 190). Both events may also involve the objectification and medicalization of the body as a thing to be measured and monitored. Thematically, both a heart attack and childbirth can also be described as involving a strange tonality, as Aho writes: “I became aware of my body as an object, as something foreign and strange. Every pinch in my chest, every constricted breath and skipped heartbeat pulled me away from the ‘I can’ and injected doubt and worry into everything I did, resulting in a profound alteration to the structures of meaning that constitute who I am” (2019: 191).

In this respect, both events also involve prepersonal bodily processes, which are not “authored” by living subjects. In the case of the heart attack, the body responds in a particular way—vomiting and nausea, as Aho notes—that may be mysterious from a lived perspective (2019: 188). Both a heart attack and childbirth involve the explicit materialization of processes that are ostensibly “hidden” from perceptual awareness in non-pathological modes of embodied life, as Aho again writes: “With my heart attack, the transparent functions of the body schema collapsed, and the corporeal body emerged out of its hiddenness” (2019: 191). The same is no less true of childbirth: birth brings to light in the most dramatic way possible the anonymous infrastructure enabling perceptual experience to operate in the first instance.

We see, then, that there are a series of similarities between childbirth and an event such as a heart attack. Yet one key difference between childbirth and an event such as a heart attack is in their overarching teleological and meaningful aims. In a word, while childbirth is oriented toward a fundamentally benevolent and generative end—namely, the creation of a child—the teleological direction of a heart attack, by contrast, is directed toward a movement of constriction without tangible benefits. Indeed, as Aho describes it in his incisive essay, the only “gift” a heart attack can bring are secondary ones that stem from an enriched sense of compassion for suffering, a renewed sense of appreciation for loved ones, and a recognition of one’s vulnerability (2019: 199). Yet these “gains” do not belong to the nature or structure of a heart attack but are instead cultivated from a relationship one has to one’s own suffering largely after the event. Childbirth, by contrast, is normally oriented toward a benign event, even though that event does not always go according to plan and can also involve prolonged suffering and sickness.

Consider here how the sense of relief described in many accounts of childbirth tends not to populate accounts of heart attacks. The reason for this is laid out in Aho’s reflections on his heart attack: the affective weight of the heart attack is not localised to the “attack” itself as though a heart attack were a discrete event easily curtailed. Rather, the attack lingers, causing a series of ripples in the structure of embodiment, spatiality, and, temporality (2019: 192). In effect, the onset of a heart attack obligates the patient to revise the entirety of their existence as they live alongside the perpetual threat of another attack. By contrast, the anxiety accompanying childbirth is broadly circumscribed to the event itself. Of course, there are other modes of anxiety that emerge in the light of birth, but these operate on another phenomenological horizon and have an indefinite variety of expressions.

There is a lot to be said here on the function of pain in relationship to a broader sense of meaning that is beyond the scope of the current paper. Questions such as whether pain can be justified, for how long it will last, and who is responsible for inflecting pain are all critical in this discussion. Yet the pain involved in childbirth and a heart attack are manifestly different insofar as the sole function of childbirth pain is to generate a specific result, whereas the “meaning” of pain in a heart attack is for it to stop (Aho 2019: 200).

There is one final point to make here concerning the relationship between childbirth and related bodily phenomena; namely, that other bodily experiences exist that involve the presentation of anonymous bodily processes without those processes being perceived as threats to the stability of selfhood. Here, for example, we can think of various forms of bodily practices and subjective experiences which involve a similar set of thematic structures to childhood—especially the sense of the body becoming strange, unfamiliar, and even taking over—without those processes causing anxiety. Examples here would include experiences that involve either the transformation or intensification of the felt body in its materiality.

Thus, eroticism—even in the case of sadomasochism (see Newmahr 2011)—would be one illustration where the body is experienced in a heightened state of arousal, such that one can sometimes have the sense of being swept up in a co-constituted rhythm that is not strictly of one’s own making.^2^ In such a case, the affective experience of pleasure is explicitly tied up with an exposure to an aspect of bodily life that is for the most part concealed by everyday familiarity.

Likewise, drug use is another example where the body can be experienced in an altered state—even as wholly absent or immaterial—without it necessarily invoking a sense of anxiety to the subject.^3^ Indeed, one aspect at work in the affective experience of pleasure involved in drug use is a movement of escape; escape not only from the surrounding world as a site of pressure and anxiety but also from the body as a zone of materiality inextricably tied up with a sense of self (see Schalow 2017). Moreover, even if the body remains fully present, then its transformation from a site of habitual normalcy to something strange and foreign can sometimes be met with fascination rather than horror (see Becker 1953). Examples such as these accent how the experience of the body in its anonymity is not localised to childbirth, nor is it necessarily shaped by the affective tonality of anxiety and panic. Rather, it permeates many aspects of perceptual life, ranging from the typical to the atypical, and which can be interpreted in a wide range of ways contingent on a multiplicity of factors.

What, finally, then does childbirth tell us about the structure of bodily life more generally? There are at least two insights here, each of which merits brief explication. First, childbirth sheds light on the ambiguous structure of embodied existence. Here, childbirth underscores how “ambiguity” not only refers to the reversibility between the body as a thing and the body as zero point of experience but also how the body can appear for us irreducibly as one’s own while also never being entirely possessable by the subject. In this respect, childbirth does not introduce a novel facet into bodily existence, but instead amplifies in an especially striking way what was there all along; namely, a level of prepersonal and anonymous existence which subtends to personal life without ever being strictly identifiable with that life. To this end, childbirth sheds light on the rich and complex structure of bodily existence, and, as the narratives in this paper testify, problematizes the pregiven and taken-for-granted idea of the body as irreducibly “one’s own”.

The second insight that childbirth generates relevant to broader bodily existence is that the concept of anonymity is not an innocuous structure of lived experience, but instead one that is laden with affective value. In much of the phenomenological literature on the body, anonymity is presented as a structural property of perceptual life, which simply reinstates the integrity of perceptual existence. But much of this literature is taken from the standpoint of a neutral subject somehow stripped of an affective atmosphere. Childbirth—but also depression, anxiety, melancholia, and several other affective states—bring to light the complex ways in which bodily life is always already mediated through our affective and circumstantial horizons. Against this context, the different levels of bodily existence operating through both childbirth and everyday life remind us that no matter how much we feel at home within our bodies, there is always an element of us that remains perpetually *strange*.

## Footnotes

1 This research was funded by the Austrian Science Fund (FWF) (P33428) and the University of Vienna, which is herein gratefully acknowledged. My thanks to Audrey Petit-Trigg, Christoph Durt, Heidi Wilm, and Luna Dolezal for their encouragement on this paper. Thanks also to the delegates of the British Society for Phenomenology Annual Conference 2019 for their remarks on an earlier version of this paper. Final thanks to the anonymous reviewers for their generous comments and constructive criticism.

2 I owe the reference to sadomasochism to the anonymous reviewer of this paper.

3 I again owe this suggestion of drug use to the anonymous reviewer of this paper.
